# Supplementary material for: Venomix: a simple bioinformatic pipeline for identifying and characterizing toxin gene candidates from transcriptomic data
Source: PeerJ. 2018 Jul 31;6:e5361. doi: 10.7717/peerj.5361 (PMC6074769; doi:10.7717/peerj.5361)
Supplement: Supplemental Information 6 [file peerj-06-5361-s006.gz › FinalOutput_1E-6/Venom_peptide_isomerase_heavy_chain_2/finaltree.pdf]

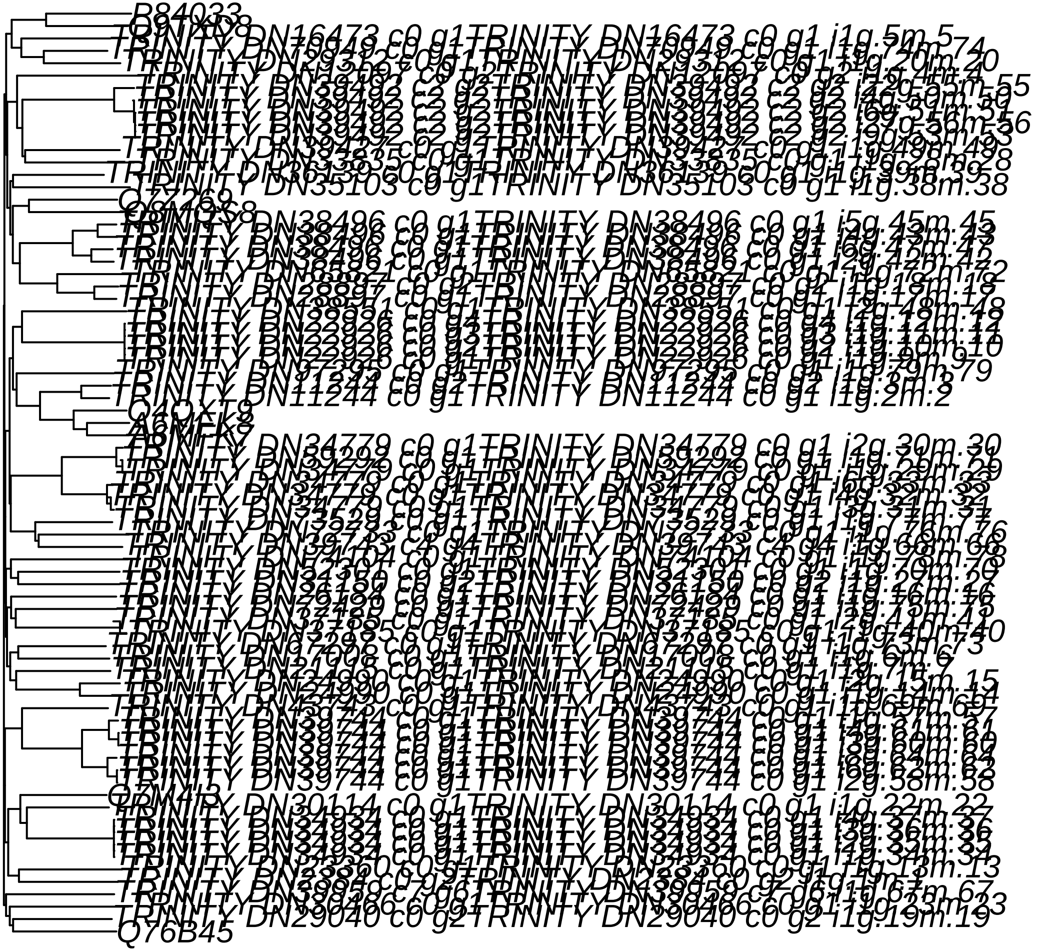
B84033
TRINITY DN16473 c0 g1TRINITY DN16473 c0 g1 i1g.5m.5
TRINITY DN35103 c0 g1TRINITY DN35103 c0 g1 i1g.38m.38
TRINITY DN38496 c0 g1TRINITY DN38496 c0 g1 i5g.45m.45
TRINITY DN11244 c0 g1TRINITY DN11244 c0 g1 i1g.2m.2
TRINITY DN34779 c0 g1TRINITY DN34779 c0 g1 i2g.30m.30
TRINITY DN39744 c0 g1TRINITY DN39744 c0 g1 i2g.58m.58
TRINITY DN30114 c0 g1TRINITY DN30114 c0 g1 i1g.22m.22
TRINITY DN29040 c0 g2TRINITY DN29040 c0 g2 i1g.19m.19
Q76B45
